# A Recipe Composed of Chinese Herbal Active Components Regulates Hepatic Lipid Metabolism of NAFLD* In Vivo* and* In Vitro*

**DOI:** 10.1155/2016/1026852

**Published:** 2016-03-16

**Authors:** Sheng-xi Meng, Qian Liu, Ya-jun Tang, Wen-jing Wang, Qing-shan Zheng, Hua-jie Tian, Dong-sheng Yao, Lin Liu, Jing-hua Peng, Yu Zhao, Yi-yang Hu, Qin Feng

**Affiliations:** ^1^Institute of Liver Diseases, Shuguang Hospital Affiliated to Shanghai University of Traditional Chinese Medicine, Shanghai 201203, China; ^2^Center for Drug Clinical Research, Shanghai University of Traditional Chinese Medicine, Shanghai 201203, China; ^3^Shanghai Key Laboratory of Traditional Chinese Clinical Medicine, Shanghai 201203, China; ^4^E-Institute of Shanghai Municipal Education Committee, Shanghai 201203, China

## Abstract

This study is to investigate the therapeutic effects of the recipe composed of* Atractylodes macrocephala* polysaccharide, chlorogenic acid, and geniposide (named ACG) on experimental nonalcoholic fatty liver (NAFL). The research was divided into two parts as screening experiment and verification experiment. In the screening experiment, we used high-fat diet (HFD) induced NAFL rat model and uniform design to get the recipe from five Chinese herbal active components. In the verification experiment, HFD induced fatty liver rat and mouse NAFL models and free fatty acid (FFA) induced HepG2 cell model were used to verify the effects of ACG. According to the multiple regression equation of the hepatic triglyceride (TG) contents of each group in the screening experiment, the recipe ACG was obtained and the doses of* Atractylodes macrocephala* polysaccharide, chlorogenic acid, and geniposide for rats were 266.67, 3.33, and 45 mg/kg, respectively. The results of verification experiment verified that ACG could significantly reduce hepatic TG contents of NAFL rats and mice, as well as the cellular TG content of FFA-induced HepG2 cells. ACG could also improve HOMA-IR and hepatic mitochondrial ultrastructure of NAFL mice. Our study verified that ACG recipe could regulate lipid metabolism of NAFL* in vivo* and* in vitro*.

## 1. Introduction

Nonalcoholic fatty liver disease (NAFLD) is characterized by excessive deposition of fat in the liver in the absence of excessive drinking of alcoholic and any secondary cause. It can develop slowly from simple nonalcoholic steatosis to nonalcoholic steatohepatitis (NASH) if inflammation is also present and subsequently to fibrosis, cirrhosis, and even hepatocellular carcinoma [1, 2]. The increasing prevalence of NAFLD has been considered as an epidemic public problem worldwide. Twenty percent to 30% of the general population in the western world suffer from NAFLD [3]. In Asia, NAFLD has been found in the range of 15% to 30% in the general population and over 50% in patients with diabetes and metabolic syndrome [4]. Studies have shown that hepatic steatosis is associated with increased mortality from cardiovascular disease, cancer, and liver disease compared with age and gender-matched populations within the same country [5].

NAFLD is considered an important public health issue, but there is currently no effective therapy. Several pharmacological agents have been studied in an effort to improve insulin resistance and the proinflammatory mediators that may be responsible for NASH progression. Vitamin E and thiazolidinedione derivatives are currently the most evidence-based therapeutic options, but only limited clinical evidence is available regarding their long-term efficacy and safety [6].

In China, traditional Chinese medicine is usually used in treating NAFLD. Qushi Huayu Decoction (QHD) is Chinese formulae which has a long history of using in clinical practice to alleviate NAFLD [7]. It consists of five herbs (*Herba Artemisiae capillaris, Polygonum cuspidatum, Hypericum japonicum* Thunb,* Gardenia*, and* Rhizoma Curcumae Longae*). The effects of QHD on experimental NAFL have been proved in previous studies [8–10]. It could inhibit hepatic lipid accumulation by activating AMP-activated protein kinase (AMPK) [10], increasing serum adiponectin, modulating HFD induced gut microbiota to a healthier structure, and so forth [11, 12].

However, due to the complicated components of traditional Chinese recipe, it is difficult to identify useful and useless ingredients in the decoction. And it is hard to control quality and be accepted by all. Since the efficacy of the recipe attributes to the active components, can we reestablish a new effective recipe only using some key active components of the traditional recipe, which has clearer ingredients and is easier to control quality? In this study, we tried to get a new effective recipe composed of QHD's active compounds and then verify its effect on NAFLD.

The study was divided into two parts as screening experiment and verification experiment. In the screening experiment, we selected five active compounds (polydatin, geniposide, chlorogenic acid, curcumin, and* Atractylodes macrocephala* polysaccharide) from QHD by means of literature review, then duplicated high-fat diet (HFD) induced NAFL rat model and used uniform design, and then got the new active components recipe. In the verification experiment, we duplicated HFD induced fatty liver rat and mouse NAFL models and free fatty acid (FFA) induced HepG2 cell model to verify the curative effect of ACG on NAFL* in vivo* and* in vitro*.

## 2. Materials and Methods

### 2.1. Uniform Design of the Screening Experiment

Uniform design was applied to screen the dosage and formula of the mixture. Uniform design tables are expressed as *U*_*n*_(*t* ^*s*^), where *U* stands for the uniform design, *n* stands for the number of experimental trials, and *t* and *s* stand for the number of levels and the maximum number of factors, respectively. In this study, the uniform design table *U*_11_(11^10^) was applied to arrange the experiments. The levels and factors are listed in Table 1. Chlorogenic acid, polydatin,* Atractylodes macrocephala* polysaccharide, geniposide, and curcumin were five factors; each factor has five different doses' levels of gradient increment and each of them repeated two times. The total is divided into ten groups (number 1~number 10) by the method of block design.

### 2.2. Drug Preparation of Screening Experiment

Chlorogenic acid (drug concentration was 98%, lot number GY0900705) derived from* Herba Artemisiae capillaris*, polydatin (drug concentration was 98%, lot number RE090703) derived from* Polygonum cuspidatum*,* Atractylodes macrocephala* polysaccharide (drug concentration was 80% UV, lot number GY090510) derived from* Rhizoma Atractylodis Macrocephalae*, geniposide (drug concentration was 98%, lot number CY101018) derived from* Gardenia*, and curcumin (drug concentration was 95% UV, lot number CU090912) derived from* Rhizoma Curcumae Longae*. All the active ingredients were purchased from Xi'an Guanyu Biotechnology Co. Ltd. Combining conventional clinical dosage of each herb and the separation rate of each active ingredient from Chinese herb, the dose range of each effective compound was determined after conversion. The doses of chlorogenic acid, polydatin,* Atractylodes macrocephala* polysaccharide, geniposide, and curcumin are ranging from 0.67 to 3.33, 1 to 5, 53.33 to 266.67, 9 to 45, and 5.5 to 27.5 mg/kg rat weight. We arranged all the factors and levels according to the uniform design table and then carried out the block design and ultimately determined the dosage of each group. The doses of each group are shown in Table 1.

### 2.3. Animal Grouping and Treatment of Screening Experiment

Sixty male Sprague Dawley (SD) rats, weighing 150 ± 10 g, were obtained from Shanghai Experimental Animal Center of Chinese Academy of Sciences, China. They were maintained under controlled temperatures (22 ± 1°C) and relative humidity (56 ± 5°C) and on a 12-hour light, 12-hour dark cycle in the animal center (Shanghai University of Traditional Chinese Medicine, Shanghai, China). All animals had free access to diet and water during the screening experiment. The study was carried out under the guidelines for animal experimentation, and the protocol was approved by the animal studies ethics committee of Shanghai University of TCM. The animal protocol number is 20130168.

After one-week acclimation, animals were randomized into 10 groups (number 1~number 10, 4 rats in each group). All the rats were fed with HFD (Shanghai Laboratory Animal, Shanghai, China; protein: 18.9%, carbohydrate: 44.6%, and fat 36.5% kcal/g). After 4 weeks, rats were dosed by oral gavage once per day for 4 weeks with active ingredients compounds from groups number 1 to number 10 listed in Table 1, respectively. At the end of the 8th week, all the animals were sacrificed and the liver was removed and stored at −70°C for subsequent analysis.

### 2.4. Evaluation of Lipid Content in Liver Tissues

Exactly 3 mL of anhydrous ethanol-acetone (1 : 1) was added to the hepatic tissues (200 mg), which was then homogenized in an ice bath and mixed thoroughly at 4°C overnight. After 12 h, the liver tissues were centrifuged at 3000 rpm and 4°C for 20 min. Subsequently, the supernatant was transferred to a new tube, and TG as the screening index was measured based on the instructions on TG assay kit (Dong'ou diagnostic products Co. Ltd., Zhejiang, China) using the colorimetric method.

### 2.5. The Method of Obtaining the Components and Doses of the Recipe ACG

After liver tissue TG data were obtained in each screening experiment group, the data were analyzed by DAS 3 software. The best effect equation was obtained by using multiple stepwise regression analysis and the statistical test with *α* = 0.05 as a significant level. When *Y* value of the regression equation is the minimum, the composition and dosage of the compound were got.

### 2.6. Verification Experiment Design

The verification experiment was divided into three parts; we compared the efficacy of the recipe ACG with the QHD on NAFLD in SD rats, C57BL/6 mice, and the fatty acid induced HepG2 cells; RGZ was selected as positive control drugs in* in vivo* experiments to verify the effects of the ACG.

### 2.7. Drug Preparation of Verification Experiment

The dosage of the recipe ACG was based on the results of the regression equation.* Atractylodes macrocephala* polysaccharide, chlorogenic acid, and geniposide for SD rats were 266.67 mg/kg, 3.33 mg/kg, and 45 mg/kg, respectively, and the dosage of C57BL/6 mice was twice that of the rats. QHD were prepared according to the previous preparation methods [10]. The five dried crude herbs of QHD were purchased from Huayu Chinese Traditional Medicine Co., Ltd. RGZ (GlaxoSmithKline Limited, Tianjin, China, lot number 10045192) was dissolved in double distilled water and made into 0.067 g/L solution. The dosage of RGZ was 0.67 mg/kg rat weight.

### 2.8. Animal Grouping and Treatment of Verification Experiment

Male SD rats (weighing 150 ± 10 g) and C57BL/6BL/6J (weighing 20 ± 3 g) mice were purchased from Shanghai Experimental Animal Center of Chinese Academy of Sciences (Shanghai, China) and kept under controlled temperatures (22 ± 1°C) and relative humidity (56 ± 5°C) and on a 12-hour light, 12-hour dark cycle. All animals had free access to diet and water during the verification experiment.

The SD rats were randomized into 5 groups: normal diet (ND) group (*n* = 9), HFD group (*n* = 9), HFD + QHD group (*n* = 9), HFD + ACG group (*n* = 9), and HFD + RGZ group (*n* = 9). Rats in the ND group were fed with 13.8% kcal fat diet (Shanghai Laboratory Animal, Shanghai, China; protein: 27.5%, carbohydrate: 60.5%, and fat 13.8% kcal/g) and the NAFLD modeling method was the same as the screening experiment. After 4 weeks, rats of medication administration teams were dosed by oral gavage once per day for 4 weeks, respectively, of 10 mL/kg/d. Furthermore, rats of ND group and HFD group were dosed by oral gavage once per day with drinking water. At the end of the 8th week, all animals were fasted overnight and sacrificed. The liver was removed and stored at −70°C for subsequent analysis. The serum was separated for further investigation.

The C57BL/6 mice were also randomized into 5 groups: ND group (*n* = 9), HFD group (*n* = 9), HFD + QHD group (*n* = 9), HFD + ACG group (*n* = 9), and HFD + RGZ group (*n* = 9). Except for the ND group mice, others were fed with HFD (Research Diets, New Brunswick; protein: 26.2%, carbohydrate: 26.3%, and fat: 34.9% kcal/g) for 12 weeks to establish the NAFLD model. From the beginning of the 9th week, mice of medication administration teams were dosed by oral gavage once per day for 4 weeks.

### 2.9. Biochemical Analysis

The alanine aminotransferase (ALT) activity, aspartate aminotransferase (AST) activity, total cholesterol (TC), triglycerides (TG), FFA, high-density lipoprotein cholesterol (HDL-c), and low-density lipoprotein cholesterol (LDL-c) of serum were determined by using commercially available kits (Jiancheng Institute of Bio Engineering, Inc., Nanjing, China) according to the manufacturer's instructions. Fasting blood glucose (FBG) was determined with a blood glucose meter (Roche diagnostic GmbH, Germany). Fasting insulin level was measured using the Mouse Insulin ELISA (ALPCO, America). The homeostasis model assessment of basal insulin resistance (HOMA-IR) was calculated using formula FBG (mM) × FIN (IU/L)/22.5. The preparation of liver homogenate was the same as the screening experiment. The hepatic TG, TC, and FFA contents were determined using commercially available kits (Dong'ou Diagnostic Products Co. Ltd., Zhejiang, China) according to the manufacturer's instructions.

### 2.10. Histological Observation

The liver sections were stained with hematoxylin and eosin (H&E) (Jiancheng Institute of Bio Engineering, Inc., Nanjing, China) and Oil-Red O (Sigma, St. Louis, MO, USA). The fresh liver tissue samples were fixed in 10% neutral formalin and embedded in paraffin. The samples were cross-cut into slices of 4 *μ*m and stained with H&E. Snap frozen tissues were placed in optimal cutting temperature compound and then sectioned and stained with Oil-Red O. All the stained sections were observed and photographed under a microscope (with 200x magnification). Disease activity was assessed with the use of the nonalcoholic fatty liver disease activity score (NAS), which is based on a standardized grading system for steatosis (on a scale of 0 to 3), lobular inflammation (on a scale of 0 to 3), and hepatocellular ballooning (on a scale of 0 to 2), with the higher score indicating as increasing severity [13].

### 2.11. Transmission Electron Microscope

The liver tissues were immediately dissected into 1 mm^3^ cubes and placed into 3% glutaraldehyde for 5 minutes at 4°C. The samples then were washed with PBS buffer 0.1 M for three times, postfixed 2 hours at room temperature in 1% osmium tetroxide, blocked for 1 hour in 3% uranyl acetate, dehydrated through graded series of ethanol and acetone, infiltrated with 100% resin for 12 hours, and embedded in the 100% resin beam capsule. Then, the samples were polymerized in the 60°C oven for 12 hours before semithin sections were done at 500 nm. Following that, the samples were stained with toluidine blue for 20 seconds and were examined under light microscope to choose the area needed for further ultrathin sectioning at 70–90 nm. The ultrathin sections were then put in the grid and then stained with uranyl acetate for 10 minutes and plumbum citrate for 5 minutes. Finally, the samples were viewed under electron microscope for lipid inclusion in the hepatocytes cytoplasm and morphology of mitochondria.

### 2.12. Preparation of Drug-Containing Serum

The 30 SPF rats, weighing 160 ± 20 g, were randomly divided into four groups. Rats in the vehicle control serum group were given 5 mL/kg of physiological saline; rats in the ACG-containing serum group were orally administered the dose received from screening experiment; rats in the QHD-containing serum group were orally administered the dose as the previous studies [9, 10]. The medication lasted 3 consecutive days (twice/day, 5 mL/kg/time). Blood was collected aseptically via the abdominal aorta 1 h after the last treatment and then centrifuged. The sera from the same group were pooled, filtered through 0.22 *μ*m filters, inactivated at 56°C for 30 min, split, and stored at −70°C for future use [14].

### 2.13. Cell Culture and Experimental Design* In Vitro*

HepG2 cells (Institutes of Biochemistry and Cell Biology, Shanghai Institutes for Biological Sciences, CAS, Shanghai, China) were cultured in DMEM supplemented with 10% heat-inactivated fetal FBS, penicillin at 100 U/mL, and streptomycin at 100 *μ*g/mL at 37°C in a 5% CO_2_ atmosphere. The experiment started when the cells grew to 70%–80% confluence. Cellular steatosis was induced by a mixture of 1 mM FFA (the ratio of oleate to palmitate is 2 : 1) in DMEM containing 1% BSA for 24 h [15]. Then HepG2 cells were divided into four groups as normal group, FFA group, FFA + 10% ACG-containing serum group (FFA + ACG), and FFA + 10% QHD-containing serum group (FFA + QHD). To ensure that the difference in the effects of the ACG-containing serum and the QHD-containing serum was attributable to the drugs not to the sera, 10% vehicle control serum was added to normal group and FFA group. After the cells were treated with the drug containing serum or vehicle control serum for another 24 h, the cells were stained with Oil-Red O and imaged.

### 2.14. Cell Oil-Red O Staining

The cells were washed with PBS twice, fixed with 10% formalin at room temperature for 10 min, and stained with a freshly prepared working solution of Oil-Red O (Sigma, St. Louis, MO, USA) at 60°C for 10 min. The cells were observed in an Olympus (Olympus Corporation, Tokyo, Japan) microscope and documented.

### 2.15. Measurement of Cellular TG Content

Total lipids in the cells and the medium were extracted and purified by using the method by Heider and Boyett [16]. The content of TG was determined with a biochemistry assay kit (Dongou Biology Technique Co. Ltd., Zhejiang, China), according to the manufacturer's instructions, and expressed as mg/g prot.

### 2.16. Statistical Analysis

Data were expressed as mean ± standard deviation for quantitative variables, *n* (%) for categorical variables. Data were analyzed by using* t*-test or one-way analysis of variance as well as the least significant difference test and *P* < 0.05 was considered statistically significant.

## 3. Results

### 3.1. Determination of Composition and Dosage of the Active Ingredients Compound ACG

In the screening experiment, the content of triglyceride in liver tissue of rats in each group was measured (Table 2), and the stepwise regression analysis was carried out. Through these processes we get the regression equation:

(1) Y=15.083X1+5.321X2−5.186X3−16.157X4+9.35X5+17.667X3X4−8.422X1X2−6.617X3X5+16.571.

According to this equation, the prescription which can reduce the TG content of liver tissue to the maximum extent is* Atractylodes macrocephala* polysaccharide (266.67 mg/kg) + Chlorogenic acid (3.33 mg/kg) + Geniposide (45 mg/kg) and we named this composition as ACG.

### 3.2. Effects of ACG on Body Weight, Liver Weight, and Liver Index (Liver/Body Weight Ratio) of HFD Fed Rats and Mice

At the end of the last experimental week, the body weight, liver wet weight, and liver index of the SD rats in HFD group were higher than those in ND group (Table 3, *P* < 0.01). Compared with the HFD group, the body weight and liver wet weight of rats in HFD + ACG group, HFD + QHD group, and HFD + RGZ group significantly decreased (Table 3, *P* < 0.01). In addition, only in the HFD + ACG group, the liver index of rats significantly decreased compared with that in the HFD group (*P* < 0.01); those of the other groups have no significant difference compared with that of the HFD group.

The body weight, liver wet weight, and liver index of the C57BL/6 mice in HFD group significantly increased compared with those in the ND group (Table 4, *P* < 0.01). Compared with the HFD group, the body weight and liver wet weight of mice in HFD + ACG group, HFD + QHD group, and HFD + RGZ group significantly decreased (Table 4, *P* < 0.01) and the liver wet weight of the HFD + ACG group significantly decreased compared with the HFD + RGZ group (*P* < 0.01). In the same way, the liver index of mice in the HFD + ACG group significantly decreased compared with that in the HFD group (*P* < 0.01).

### 3.3. Effects of ACG on Hepatic Lipids of HFD Fed Rats and Mice

**Figure 1:**
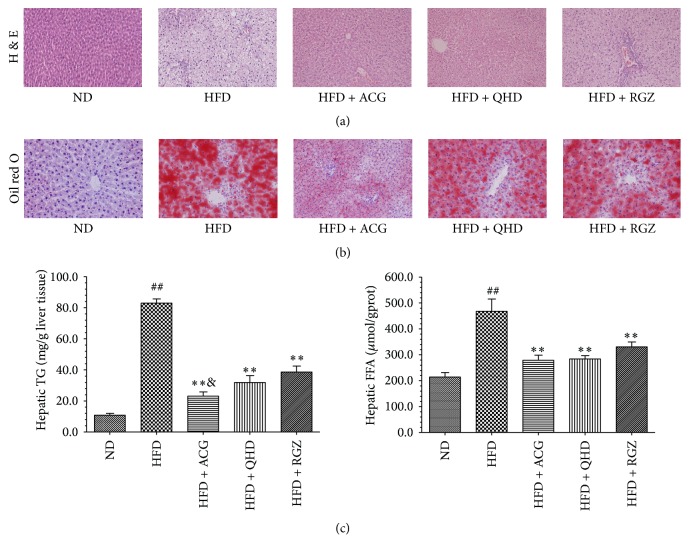
ACG ameliorates lipid accumulation in the liver of HFD induced obesity SD rats. (a) H&E staining of liver sections (×200). (b) Oil-Red O staining of liver sections (×200). (c) Effects of ACG on hepatic TG and FFA levels. The values reported in the figure represent the means ± SD (*n* = 9). ^##^ *P* < 0.01 versus the ND group; ^*∗∗*^ *P* < 0.01 versus the HFD group; ^&^ *P* < 0.05 versus the HFD + RGZ group.

In SD rats, the levels of TG and FFA in liver tissues of the HFD group significantly increased compared with those in the ND group (*P* < 0.01) (Figure 1(c)). The hepatic TG in the treatment groups significantly decreased compared with those in the HFD group (*P* < 0.01), but in the HFD + ACG group they were significantly lower than that in the HFD + RGZ group (*P* < 0.05). In addition, the hepatic FFA contents of the three treatment groups were lower than that in the HFD group (*P* < 0.01), but there was no significant difference among the groups.

**Figure 2:**
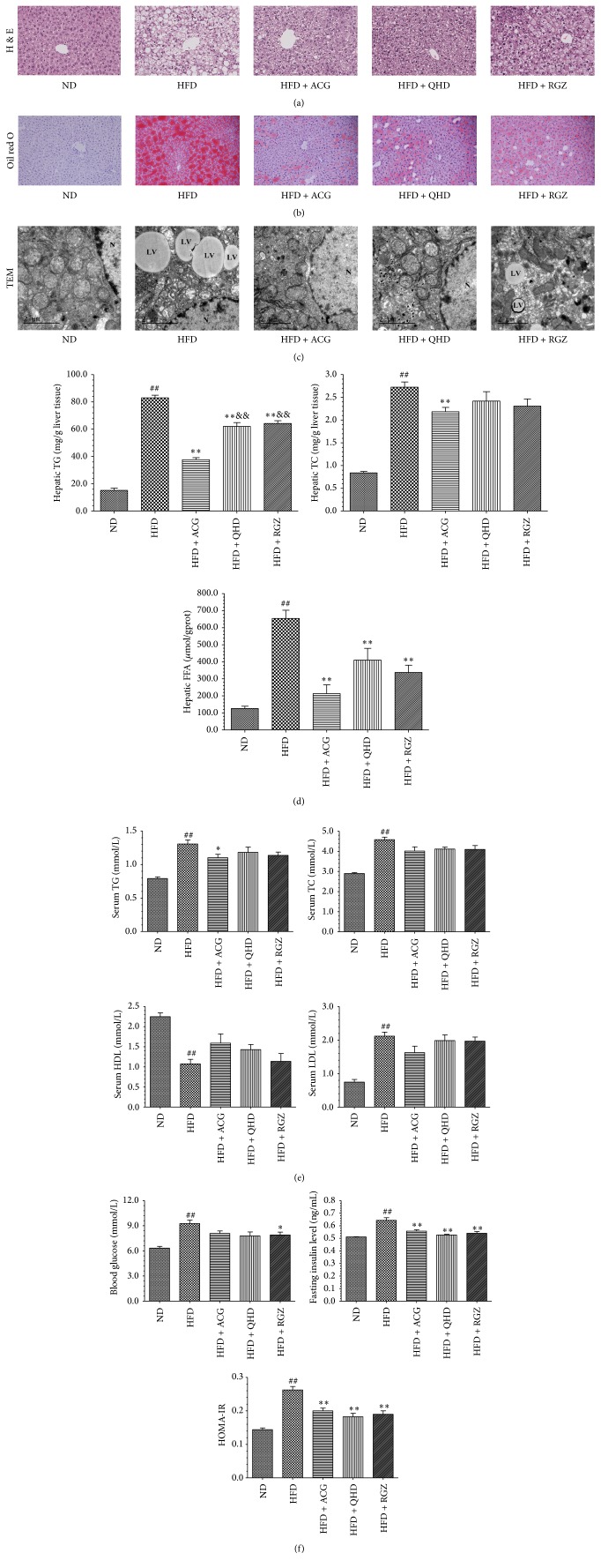
Effects of ACG on lipid metabolism and glycometabolism in HFD induced obesity C57BL/6 mice. (a) H&E staining of liver sections (×200). (b) Oil-Red O staining of liver sections (×200). (c) The transmission electron micrograph of hepatocytes at magnification ×9900. N: nucleus and LV: lipid vacuoles. (d) Hepatic TG, TC, and FFA levels. (e) Serum lipid: TG, TC, HDL, and LDL. (f) Blood glucose, insulin level, and HOMA-IR. The values reported in the figure represent the means ± SD (*n* = 9). ^*∗*^ *P* < 0.05 versus the HFD group; ^##^ *P* < 0.01 versus the ND group; ^*∗∗*^ *P* < 0.01 versus the HFD group; ^&&^ *P* < 0.01 versus the HFD + ACG group.

Similarly, ACG treatment also decreased hepatic TG and FFA concentration of C57BL/6 mice when compared to HFD group, and the effect of ACG on TG was more significant than that of RGZ and QHD (*P* < 0.01) (Figure 2(d)). Moreover, the results of the cholesterol content of liver tissue in C57BL/6 mice also showed that the cholesterol content of the HFD + ACG group was significantly lower than that in the HFD group (*P* < 0.01) (Figure 2(d)). The data showed that ACG treatment ameliorated hepatic steatosis significantly.

### 3.4. Effects of ACG on Serum Lipids Content and ALT, AST Activities of HFD Fed Rats and Mice

Compared with the ND group of SD rats, serum ALT and AST activities significantly increased in HFD group (*P* < 0.01 or *P* < 0.05). HFD + ACG group and HFD + QHD group showed lower ALT, AST levels and HFD + RGZ group only decreased AST when compared with the HFD group (*P* < 0.01) (Table 3). The serum ALT and AST activities significantly increased in HFD group compared with the ND group of C57BL/6 mice (*P* < 0.01); ALT, AST levels only decreased in HFD + ACG group compared with the HFD group (*P* < 0.01) (Table 4). These results suggest that ACG could protect the liver injury induced by the HFD feeding.

On the serum lipid level, serum TG, TC, and LDL-C levels significantly increased and serum HDL-C level decreased in the HFD group mice (*P* < 0.01) (Figure 2(e)) compared with the ND group C57BL/6 mice. Although there was no notable difference in serum TC, LDL-C, and HDL-C among the three treatment groups, the serum TG in the HFD + ACG group was lower than that in the HFD group (*P* < 0.05) (Figure 2(e)).

### 3.5. Effects of ACG on Blood Glucose, Insulin Level, and HOMA-IR of C57BL/6 Mice

The blood glucose level reduced significantly only in C57BL/6 mice after medicating with RGZ (*P* < 0.05) (Figure 2(f)), while medication with ACG or QHD did not influence the level of blood glucose of the animals. Insulin levels and HOMA-IR were significantly reduced in three treatment groups following the HFD and there was no significant difference between HFD + ACG group, HFD + QHD group, and HFD + RGZ group. These results suggest that the effect of ACG or QHD on blood glucose is not significant, but it has a certain effect on the level of insulin and insulin resistance.

### 3.6. Effects of ACG on Liver Histopathology

The histological changes in liver samples were examined in H&E and Oil-Red O stained sections. H&E staining of liver tissues in both SD rats and C57BL/6 mice showed that the liver samples in the HFD group had different degrees of fatty degeneration, hepatocyte ballooning, punctiform necrosis, and leukocyte infiltration. Compared with the HFD group, the degree of steatosis and inflammation of liver samples in HFD + ACG group were significantly decreased (Figures 1(a) and 2(a)). Histological examination with Oil-Red O staining showed that ACG treated animals' livers had significantly less fat deposition in hepatocytes when compared to those of HFD group rats and mice (Figures 1(b) and 2(b)).

Average score of histopathological findings in liver samples of SD rats showed that the degree of hepatic steatosis and ballooning in HFD group was more serious than that in ND group (*P* < 0.01). The degree of hepatic steatosis and ballooning in HFD + ACG group and HFD + QHD group decreased significantly compared to that in HFD group (*P* < 0.01). However, the inflammation in each group's rats is not prominent (Table 5).

Average score of histopathological findings in livers of C57BL/6 mice showed that the score of fatty degeneration and inflammation of liver significantly increased in HFD group compared with the ND group (*P* < 0.01) (Table 6). The degree of liver steatosis in HFD + ACG group, HFD + QHD group, and HFD + RGZ group significantly decreased compared to that in HFD group (*P* < 0.01 or *P* < 0.05). The degree of inflammation and ballooning in HFD + ACG group and HFD + QHD group was less than that in HFD group (*P* < 0.01).

### 3.7. Effects of ACG on Hepatic Steatosis and Mitochondrial Ultrastructure

The hepatic steatosis with evidence of deposition of lipid vacuoles in the cytoplasm of hepatocyte is shown in Figure 2(c). In HFD group, majority of lipid vacuoles were arranged either in singlet or in groups of 2-3 vacuoles and the nuclei of them were pushed to the periphery. In the ND group, the size of mitochondria ranged between 0.5 and 2 *μ*m and the cristae of the mitochondria were well defined and arranged closely to one another. In contrast, the mitochondria cristae in the HFD group were disrupted and sparse between one another and the matrix was decreased in density or hypodense. The distribution density of fat in HFD + ACG group and HFD + QHD group was lower than that of HFD group, and the abnormal ultrastructure of mitochondria was lighter than that of HFD group (Figure 2(c)).

### 3.8. Effects of ACG-Medicated Serum on FFA-Induced Cellular Lipid Steatosis and TG Content in HepG2 Cells

**Figure 3:**
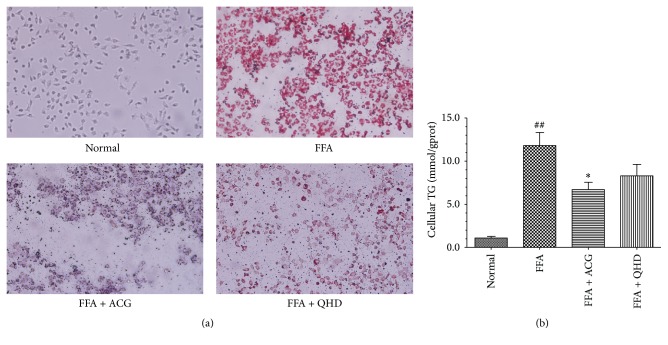
Effects of ACG on HepG2 cellular steatosis. (a) The cells were stained with Oil-Red and observed under microscope (×200). (b) ACG reduces intracellular triacylglycerol accumulation in HepG2 cells. The values reported in the figure represent the means ± SD (*n* = 9). ^##^ *P* < 0.01 versus the normal group; ^*∗*^ *P* < 0.05 versus the FFA group.

The HepG2 cells in the normal group had almost no steatosis, whereas severe steatosis and macrolipid droplets were observed in the FFA group. The number and size of the lipid droplets were reduced in the FFA + 10% ACG-containing serum group and the FFA + 10% QHD-containing serum group compared with those in the FFA group (Figure 3(a)).

Furthermore, the intracellular TG content increased significantly in the FFA group compared with that in the normal group (*P* < 0.01) and decreased significantly in the FFA + 10% ACG-containing serum group (*P* < 0.05 versus the FFA group).

## 4. Discussion

There is still no satisfactory treatment for NAFLD. Use of currently available antihyperlipidemia, hypoglycemia, or anti-inflammation drugs to treat NAFLD has not achieved desirable outcomes. A growing attention thus has been paid towards natural products as alternative means in treating NALFD.

QHD, a Chinese herbal formula, has been proven to be effective on alleviating NAFLD in human and rats [7, 17]. Previous studies also showed that its pharmacological mechanism is related to activating AMPK, reducing tumor necrosis factor-*α* (TNF-*α*) expression, regulating the intestinal flora of disbalance, and so forth [9–11]. QHD containing several herbs to ameliorate a set of abnormalities is a special feature of traditional Chinese medicine formula. But the complicated composition is difficult to understand and control. To avoid the limitations, we tried to explore a new effective recipe, which was composed of several clear and definite Chinese herbal compounds. If the new recipe has similar curative effect compared to the traditional formula, it would be much better due to its advantages of clear constituent and quality stability.

QHD contains five herbs and each herb has many specific and unspecific ingredients. Which compounds should we select from so many active ingredients? How could we reorganize the new recipe? Our research team successfully launched several studies on Chinese herbal active components recipes by using the mathematical model of uniform design which was proposed by Fang [18–20].

Uniform design is an experimental design originated from number theory and multivariate statistical. The design makes each test point more representative and then reduces the number of trials. So it is suitable for multifactor and multiple level tests. Meanwhile, it can also be used to analyze the effect of various experimental factors and search for the optimal experimental parameters by using multiple stepwise regression analysis [19].

In the screening experiment of our study, the uniform design table *U*_11_(11^10^) was applied to arrange the experiments. We selected chlorogenic acid, polydatin,* Atractylodes macrocephala* polysaccharide, geniposide, and curcumin, which is, respectively, main active compound of five Chinese herbs in QHD according to the Chinese pharmacopoeia and related literature reports. Because NAFLD is characterized by the accumulation of TGs in liver, the hepatic TG content is the main items of NAFLD. The hepatic TG content was selected as the screening index during screening new active compounds recipe. The equation was obtained from multiple stepwise regression analysis of each group's TG content. According to the equation, when* Atractylodes macrocephala* polysaccharide, chlorogenic acid, and geniposide combined together according to the proportion of 266.67 : 3.33 : 45, the hepatic TG content would be lowest. Therefore, we organized the three compounds together as the new recipe ACG.

After the active components recipe being obtained, we further need to validate whether ACG is effective on NAFLD and whether its pharmacological effect is similar to QHD. So in the following verification experiment, HFD induced fatty liver rat and mouse NAFL models and FFA-induced HepG2 cell model were duplicated to verify the effects of ACG* in vivo* and* in vitro*. RGZ was selected as positive control drugs in* in vivo* experiments.

The results of high-fat induced NALF models of rat and mouse demonstrated that the treatment of the ACG, QHD, and RGZ all blocked the body weight gain induced by HFD, while the food intake of SD rats was not changed markedly, indicating that the effect is not caused by a decrease of calorie intake. The lipid deposition degree in the liver is the main reviewing items of NAFLD. The results of both rats and mice showed that ACG had similar effects to QHD on decreasing the hepatic TG and FFA. HE staining and Oil-Red staining results showed that the ACG obviously improved fatty deposits and inflammation of liver tissue. The average activity scores of HFD + ACG group in rats and mice also declined. All these results indicated that the administration of ACG can dramatically inhibit hepatic lipid deposition and inflammation. Twelve weeks of HFD feeding in C57BL/6 mice caused dyslipidaemia with high levels of serum TG, low levels of HDL-C, and elevations in LDL-C concentrations. ACG could decrease serum TG significantly.

In order to further study the effect of ACG on lipid deposition in hepatocytes* in vitro*, HepG2 cells were incubated with a mixture of FFA (oleic acid : palmitic acid at 2 : 1) to induce cellular steatosis. The results showed that 10% ACG-containing serum could significantly reduce the accumulation of lipid droplets and inhibit the increasing of intracellular TG content induced by FFA. In previous study, we confirmed that the doses of 5% and 10% (vol/vol) QHD-containing serum are safe based on the MTT assay* in vitro* and 10% QHD-containing serum could inhibit cellular TG content and alleviate cellular fatty drops significantly [9]. This is the reason why we still used the 10% QHD-containing serum in the present study. The results of the* in vitro* experiment demonstrated that ACG might have a direct effect on hepatocellular lipid metabolism.

Thiazolidinediones currently in clinical use include rosiglitazone and pioglitazone. This class of oral medications works by activating the peroxisome proliferator-activated receptor *γ* (PPAR-*γ*), a nuclear receptor expressed in the liver, muscle, and adipose tissue that regulates adipocyte differentiation, fat metabolism, and inflammation. Activation of the receptor leads to multiple potential beneficial effects for NAFLD including inhibiting hepatic fatty acid synthesis [21], remodeling adipose tissue to sequester fatty acids [22], and promoting an insulin sensitive profile by increasing adiponectin levels [23]. Therefore, we chose RGZ as a positive control drug in* in vivo* experiment. Specifically, ACG treatment has more favorable effects on hepatic TG than RGZ. In addition, the effect of ACG on fasting insulin levels and HOMA-IR of C57BL/6 mice is also similar to RGZ. The results indicated that the inhibitory effect of ACG on hepatic lipid deposition may be related to the improvement of insulin sensitivity in C57BL/6 mice and the concrete action mechanism is worth further study.

To further investigate the potential influencing of ACG on the energy metabolism of hepatocytes, the changes in the structure of mitochondria in NAFLD C57BL/6 mice by using TEM were observed also. The mitochondria in HFD group of C57BL/6 mice showed morphological disruption whereby the sizes were increased, the cristae were disrupted, and the matrix was hypodense compared to the ND group. However, these changes in the QHD and ACG groups were less than those in the HFD group. The microscopic morphology observed by TEM indicated that ACG might have protective effect on the mitochondrial dysfunction induced by HFD, which is also consistent with the effect of inhibiting the hepatic steatosis. Therefore, the mechanism of ACG on mitochondria is worthy of further study.

As expected, the active ingredients compound in ACG can inhibit hepatic lipid deposition and hepatocellular inflammation as well as QHD. As we know, herbal active ingredients contain multiple naturally occurring compounds that can target different pathological pathways involved in the disease, providing therapeutic effects via a spectrum of actions. Other previous experimental studies show that polysaccharides have the effects of antioxidant, reducing the damage of viral liver and improving the immune function [24]. Furthermore, it has protective and therapeutic effect of ischemia reperfusion injury on liver in rats, which may be related to its antioxidant properties and inhibition of the activity of nuclear factor *κ*B [25]. Geniposide, which is iridoid glycoside from the fruit of* Gardenia jasminoides* Ellis, is recognized as being useful against hyperlipidemia and fatty liver and the mechanism may be related to the antioxidant properties and the expression of PPAR-*α* [26]. In addition, the liver protective effect of geniposide may be related to its inhibition of the activity of cytochrome P450s and promoting effect of the expression of glutathione-S-transferase gene [27]. Chlorogenic acid, a major constituent in* Artemisia capillaris* and green coffee bean and so forth, has shown a significant influence on glucose metabolism [28, 29]. Moreover, chlorogenic acid has the effect of lowering cholesterol and improving the fatty liver in rats induced by high cholesterol diet through upregulating the expression of PPAR-*α* and the increase of the fatty acid unit [30]. These previous studies have indicated that the three active ingredients of ACG have positive effects on the liver, and some pharmacological studies results may be used as references for the further study of the mechanism. Interestingly, the dose proportion of* Atractylodes macrocephala* polysaccharide, geniposide, and chlorogenic acid in ACG was 89 : 15 : 1, so how do they work together? All these need to be further explored in the later research.

## 5. Conclusion

In conclusion, the results of the present study show that recipe ACG can inhibit the hepatic lipid deposition and has good therapeutic effect on NAFLD. Further studies are needed to be carried out to evaluate the mechanism involved in reversal of hepatic steatosis.

## Figures and Tables

**Table 1:** The amount of active ingredients in each group of screening experiment (mg/kg/d).

Groups	Chlorogenic acid (*X*_1_)	Polydatin (*X*_2_)	Atractylodes polysaccharide (*X*_3_)	Gardenoside (*X*_4_)	Curcumin (*X*_5_)
Number 1	0.67	1.00	106.67	27.00	22.00
Number 2	0.67	2.00	160.00	45.00	11.00
Number 3	1.33	3.00	106.67	18.00	27.50
Number 4	1.33	4.00	53.33	45.00	16.50
Number 5	2.00	5.00	266.67	18.00	5.50
Number 6	2.00	1.00	213.33	36.00	27.50
Number 7	2.67	2.00	266.67	9.00	16.50
Number 8	2.67	3.00	53.33	36.00	5.50
Number 9	3.33	4.00	160.00	9.00	22.00
Number 10	3.33	5.00	213.33	27.00	11.00

**Table 2:** The TG content of liver tissue in each group of rats in the screening experiment.

Groups	TG (mg/g liver tissue)
Number 1	41.66 ± 9.14
Number 2	29.90 ± 8.48
Number 3	43.13 ± 22.36
Number 4	73.26 ± 10.00
Number 5	48.30 ± 15.81
Number 6	31.30 ± 24.81
Number 7	42.14 ± 9.18
Number 8	69.34 ± 33.37
Number 9	40.25 ± 17.55
Number 10	39.45 ± 9.79

Data are expressed as means ± SD (*n* = 4; “*n*” means the rat number of each group).

**Table 3:** Body weight, food intake, liver weight, liver/body weight ratio, and serum transaminase in different groups of SD rats.

Parameters^a^	ND	HFD	HFD + ACG	HFD + QHD	HFD + RGZ
Body weight (g)	438.33 ± 23.96	588.89 ± 52.05^##^	478.67 ± 56.05^*∗∗*^	484.22 ± 58.47^*∗∗*^	495.22 ± 44.61^*∗∗*^
Food intake (g/day)	19.65 ± 2.2	21.53 ± 2.6	20.82 ± 2.3	20.00 ± 2.1	21.44 ± 2.2
Liver weight (g)	12.82 ± 1.36	23.06 ± 3.68^##^	16.23 ± 1.23^*∗∗*^	18.24 ± 2.13^*∗*^	18.92 ± 2.64^*∗*^
Liver/body weight ratio	2.92 ± 0.26	3.89 ± 0.34^##^	3.42 ± 0.41^*∗*^	3.74 ± 0.40	3.81 ± 0.32
ALT (U/L)	25.63 ± 11.23	39.24 ± 14.10^#^	26.42 ± 8.68^*∗*^	21.45 ± 7.85^*∗∗*^	37.56 ± 8.97
AST (U/L)	22.87 ± 4.52	39.34 ± 14.40^##^	26.11 ± 8.27^*∗*^	23.29 ± 4.91^*∗∗*^	27.13 ± 2.37^*∗*^

^a^Data are expressed as mean ± SD (*n* = 9).

Statistical analysis of the data was performed using the one-way ANOVA test followed by Tukey's *post hoc *test.

^##^ *P* < 0.01 versus the ND group.

^#^ *P* < 0.05 versus the ND group.

^*∗∗*^ *P* < 0.01 versus the HFD group.

^*∗*^ *P* < 0.05 versus the HFD group.

**Table 4:** Body weight, food intake, liver weight, liver/body weight ratio, and serum transaminase indifferent groups of C57BL/6 mice.

Parameters^a^	ND	HFD	HFD + ACG	HFD + QHD	HFD + RGZ
Body weight (g)	29.69 ± 2.89	45.85 ± 2.23^##^	34.69 ± 2.84^*∗∗*^	32.75 ± 2.66^*∗∗*^	33.54 ± 2.57^*∗∗*^
Food intake (g/day)	2.60 ± 0.09	2.77 ± 0.14	2.65 ± 0.10	2.62 ± 0.08	2.73 ± 0.08
Liver weight (g)	1.06 ± 0.10	2.45 ± 0.14^##^	1.35 ± 0.38^*∗∗*&^	1.56 ± 0.45^*∗∗*^	1.67 ± 0.43^*∗∗*^
Liver/body weight ratio	3.60 ± 0.37	5.36 ± 0.30^##^	3.95 ± 1.39^*∗*^	4.78 ± 1.52	5.04 ± 1.46
ALT (U/L)	30.31 ± 7.54	91.65 ± 20.78^##^	55.18 ± 13.66^*∗∗*^	71.98 ± 19.55	81.68 ± 20.5
AST (U/L)	20.42 ± 4.26	87.83 ± 18.26^##^	60.46 ± 13.51^*∗∗*^	74.84 ± 11.91	82.44 ± 16.76

^a^Data are expressed as mean ± SD (*n* = 9).

Statistical analysis of the data was performed using the one-way ANOVA test followed by Tukey's *post hoc *test.

^##^ *P* < 0.01 versus the ND group.

^*∗∗*^ *P* < 0.01 versus the HFD group.

^*∗*^ *P* < 0.05 versus the HFD group.

^&^ *P* < 0.05 versus the HFD + RGZ group.

**Table 5:** Average score of histopathological findings in livers of SD rats.

Parameters^a^	ND	HFD	HFD + ACG	HFD + QHD	HFD + RGZ
Steatosis	0 ± 0	2.56 ± 0.53^##^	1.56 ± 0.53^*∗∗*^	1.56 ± 0.53^*∗∗*^	2.0 ± 0.50
Inflammation	0 ± 0	0.56 ± 0.53	0.22 ± 0.44	0.22 ± 0.44	0.33 ± 0.5
Ballooning	0 ± 0	1.89 ± 0.33^##^	1.11 ± 0.33^*∗∗*^	1.22 ± 0.44^*∗∗*^	1.44 ± 0.52

^a^Data are expressed as mean ± SD (*n* = 9).

Statistical analysis of the data was performed using the one-way ANOVA test followed by Tukey's *post hoc *test.

^##^ *P* < 0.01 versus the ND group.

^*∗∗*^ *P* < 0.01 versus the HFD group.

**Table 6:** Average score of histopathological findings in livers of C57BL/6 mice.

Parameters^a^	ND	HFD	HFD + ACG	HFD + QHD	HFD + RGZ
Steatosis	0 ± 0	2.67 ± 0.50^##^	1.67 ± 0.71^*∗∗*^	1.56 ± 0.53^*∗∗*^	1.89 ± 0.60^*∗*^
Inflammation	0 ± 0	1.22 ± 0.97^##^	0.44 ± 0.53^*∗*^	0.56 ± 0.52	0.89 ± 0.33
Ballooning	0 ± 0	2.00 ± 0^##^	1.22 ± 0.83^*∗*^	1.33 ± 0.50^*∗*^	1.56 ± 0.53

^a^Data are expressed as mean ± SD (*n* = 9).

Statistical analysis of the data was performed using the one-way ANOVA test followed by Tukey's *post hoc *test.

^##^ *P* < 0.01 versus the ND group.

^*∗∗*^ *P* < 0.01 versus the HFD group.

^*∗*^ *P* < 0.05 versus the HFD group.

## References

[1] Cohen J. C., Horton J. D., Hobbs H. H. (2011). Human fatty liver disease: old questions and new insights. *Science*.

[2] Targher G., Chonchol M., Pichiri I., Zoppini G. (2011). Risk of cardiovascular disease and chronic kidney disease in diabetic patients with non-alcoholic fatty liver disease: just a coincidence?. *Journal of Endocrinological Investigation*.

[3] Lazo M., Hernaez R., Eberhardt M. S. (2013). Prevalence of nonalcoholic fatty liver disease in the United States: the third national health and nutrition examination survey, 1988–1994. *American Journal of Epidemiology*.

[4] Ashtari S., Pourhoseingholi M. A., Zali M. R. (2015). Non-alcohol fatty liver disease in Asia: prevention and planning. *World Journal of Hepatology*.

[5] Adams L. A., Lymp J. F., St Sauver J. (2005). The natural history of nonalcoholic fatty liver disease: a population-based cohort study. *Gastroenterology*.

[6] Milić S., Mikolasevic I., Krznaric-Zrnic I. (2015). Nonalcoholic steatohepatitis: emerging targeted therapies to optimize treatment options. *Drug Design, Development and Therapy*.

[7] Mu Y. P., Du J., Liu P. (2009). Herbal prescription experience in the treatment of non-alcoholic steatohepatitis of Professor Liu Ping. *Chinese Journal of Integrated Traditional and Western Medicine on Liver Diseases*.

[8] Zhang H., Feng Q., Li H.-S. (2008). Effects of Qushi Huayu Decoction on cathepsin B and tumor necrosis factor-*α* expression in rats with non-alcoholic steatohepatitis. *Zhong Xi Yi Jie He Xue Bao*.

[9] Feng Q., Cheng Y., Hu Y.-Y., Zhang H., Peng J.-H., Zhang N. (2010). Qushi Huayu Decoction (fb) inhibits protein and gene expression of cathepsin B in HepG2 cells induced by free fatty acids. *Chinese Journal of Integrative Medicine*.

[10] Feng Q., Gou X.-J., Meng S.-X. (2013). Qushi huayu decoction inhibits hepatic lipid accumulation by activating AMP-activated protein kinase in vivo and in vitro. *Evidence-Based Complementary and Alternative Medicine*.

[11] Yin X., Peng J., Zhao L. (2013). Structural changes of gut microbiota in a rat non-alcoholic fatty liver disease model treated with a Chinese herbal formula. *Systematic and Applied Microbiology*.

[12] Li H.-S., Feng Q., Hu Y.-Y. (2009). The role of adiponectin and adiponectin receptor 2 in the pathology of fatty liver. *Zhonghua Gan Zang Bing Za Zhi*.

[13] Kleiner D. E., Brunt E. M., Van Natta M. (2005). Design and validation of a histological scoring system for nonalcoholic fatty liver disease. *Hepatology*.

[14] Meng Z.-Z., Hu J.-H., Chen J.-X., Yue G.-X. (2012). Xiaoyaosan decoction, a traditional Chinese medicine, inhibits oxidative-stress-induced hippocampus neuron apoptosis in vitro. *Evidence-Based Complementary and Alternative Medicine*.

[15] Gómez-Lechón M. J., Donato M. T., Martínez-Romero A., Jiménez N., Castell J. V., O'Connor J.-E. (2007). A human hepatocellular in vitro model to investigate steatosis. *Chemico-Biological Interactions*.

[16] Heider J. G., Boyett R. L. (1978). The picomole determination of free and total cholesterol in cells in culture. *Journal of Lipid Research*.

[17] Li H.-S., Feng Q., Hu Y.-Y. (2009). Effect of qushl huayu decoction on high-fat diet induced hepatic lipid deposition in rate. *Zhongguo Zhong Xi Yi Jie He Za Zhi*.

[18] Fang K. T. (1980). Uniform design: an application of number-theoretic methods to experimental designs. *Acta Mathematicae Applicatae Sinica*.

[19] Xu W. J., Zhou H. H., Chen S. D. (2010). Uniform design research on complex prescription of Chinese medicine. *Chinese Journal of Experimental Traditional Medical Formulae*.

[20] Fu Q.-L., Hu Y.-Y., Feng Q., Wang X.-N., Peng J.-H., Cui T. (2011). Analysis of major herbs in Chinese herbal formula Jianpi Huoxue Decoction for improving intestinal permeability based on uniform design. *Zhong Xi Yi Jie He Xue Bao*.

[21] Browning J. D., Horton J. D. (2004). Molecular mediators of hepatic steatosis and liver injury. *Journal of Clinical Investigation*.

[22] Semple R. K., Chatterjee V. K. K., O'Rahilly S. (2006). PPAR*γ* and human metabolic disease. *Journal of Clinical Investigation*.

[23] Monsalve F. A., Pyarasani R. D., Delgado-Lopez F., Moore-Carrasco R. (2013). Peroxisome proliferator-activated receptor targets for the treatment of metabolic diseases. *Mediators of Inflammation*.

[24] Jin C., Zhang P.-J., Wu X.-M. (2009). Impact of hypoxic preconditioning on apoptosis and its possible mechanism in orthotopic liver autotransplantation in rats. *Hepatobiliary and Pancreatic Diseases International*.

[25] Cheng J., Zhang P.-J., Bao C.-Q. (2011). Protective effects of atractylodes macrocephala polysaccharide on liver ischemiareperfusion injury and its possible mechanism in rats. *American Journal of Chinese Medicine*.

[26] Ma T., Huang C., Zong G. (2011). Hepatoprotective effects of geniposide in a rat model of nonalcoholic steatohepatitis. *Journal of Pharmacy and Pharmacology*.

[27] Kuo W.-H., Wang C.-J., Young S.-C., Sun Y.-C., Chen Y.-J., Chou F.-P. (2004). Differential induction of the expression of GST subunits by geniposide in rat hepatocytes. *Pharmacology*.

[28] Vinson J. A., Burnham B. R., Nagendran M. V. (2012). Randomized, double-blind, placebo-controlled, linear dose, crossover study to evaluate the efficacy and safety of a green coffee bean extract in overweight subjects. *Diabetes, Metabolic Syndrome and Obesity*.

[29] Kojima K., Shimada T., Nagareda Y. (2011). Preventive effect of geniposide on metabolic disease status in spontaneously obese type 2 diabetic mice and free fatty acid-treated HepG2 cells. *Biological and Pharmaceutical Bulletin*.

[30] Wan C.-W., Wong C. N.-Y., Pin W.-K. (2013). Chlorogenic acid exhibits cholesterol lowering and fatty liver attenuating properties by up-regulating the gene expression of PPAR-*α* in hypercholesterolemic rats induced with a high-cholesterol diet. *Phytotherapy Research*.

